# Multi-view graph convolutional network for cancer cell-specific synthetic lethality prediction

**DOI:** 10.3389/fgene.2022.1103092

**Published:** 2023-01-09

**Authors:** Kunjie Fan, Shan Tang, Birkan Gökbağ, Lijun Cheng, Lang Li

**Affiliations:** ^1^ Department of Biomedical Informatics, The Ohio State University, Columbus, OH, United States; ^2^ College of Pharmacy, The Ohio State University, Columbus, OH, United States

**Keywords:** synthetic lethality, systems biology, graph neural networks, deep learning, multi-omics

## Abstract

Synthetic lethal (SL) genetic interactions have been regarded as a promising focus for investigating potential targeted therapeutics to tackle cancer. However, the costly investment of time and labor associated with wet-lab experimental screenings to discover potential SL relationships motivates the development of computational methods. Although graph neural network (GNN) models have performed well in the prediction of SL gene pairs, existing GNN-based models are not designed for predicting cancer cell-specific SL interactions that are more relevant to experimental validation *in vitro*. Besides, neither have existing methods fully utilized diverse graph representations of biological features to improve prediction performance. In this work, we propose MVGCN-iSL, a novel multi-view graph convolutional network (GCN) model to predict cancer cell-specific SL gene pairs, by incorporating five biological graph features and multi-omics data. Max pooling operation is applied to integrate five graph-specific representations obtained from GCN models. Afterwards, a deep neural network (DNN) model serves as the prediction module to predict the SL interactions in individual cancer cells (iSL). Extensive experiments have validated the model’s successful integration of the multiple graph features and state-of-the-art performance in the prediction of potential SL gene pairs as well as generalization ability to novel genes.

## 1 Introduction

Synthetic lethal (SL) is a functional relationship between two genes where the loss of either gene is viable while the loss of both is lethal. The identification of gene pairs that demonstrate synthetic lethality can help uncover potential mechanisms that will contribute to the discovery of anti-cancer targets and development of therapeutic drugs. For example, olaparib and niraparib, two PARP inhibitors, are FDA-approved drugs used to treat breast and ovarian cancer in patients with BRCA mutations based on the well-known SL relationships between PARP and BRCA1 and BRCA2 genes (Chan and Giaccia, 2011). However, such SL gene pairs remain largely unclear in cancer cells, so the development of experimental technologies and computational methods is urgently needed for their discovery and validation.

A gene that is required for the reproductive success of a cell or an organism under a specific condition is considered essential, and several methods have been developed to identify gene essentiality. High-throughput genome-editing methods have been developed, including chemical libraries (Simons et al., 2001), RNA interference (RNAi) (Luo et al., 2009), and CRISPR-Cas9 (Du et al., 2017), to identity gene essentiality. Then SL gene pairs can be identified by comparing gene essentialities of the target gene between two cell groups with or without perturbation of the second query gene. More recently, combinatorial RNAi (Grimm and Kay, 2007) and combinatorial CRISPR (Vidigal and Ventura, 2015; Han et al., 2017; Boettcher et al., 2018; Najm et al., 2018; Zhou et al., 2020) techniques have been developed for parallel pairwise gene disturbance to systematically detect SL gene interactions. As a combinatorial screen experiment is technically limited to several hundred genes and their combinations, the primary challenge is the selection of candidate genes and gene pairs. This, in turn, will rely on a highly accurate computational approach.

Several computational approaches proposed for predicting potential SL pairs include rule-based statistical inference methods, network-based models and machine learning methods (Tang et al., 2022). Statistical inference methods, such as DAISY (data mining synthetic lethality identification pipeline) (Jerby-Arnon et al., 2014), ISLE (identification of clinically relevant synthetic lethality) (Lee et al., 2018), ASTER (analysis of synthetic lethality by comparison with tissue-specific disease-free genomic and transcriptomic data) (Liany et al., 2020a) and MiSL (mining synthetic lethals) (Sinha et al., 2017), perform statistical tests to infer SL pairs directly based on the definition of synthetic lethality. Using multi-omics profiles and genome-editing data from both cancer cell lines and cancer tumor samples, SL gene pairs are typically derived from mutation relationships in activation and essentiality between two genes. Network-based methods to select gene combinations rank the combinations based on the proportion of the network they control or regulate, frequently identifying many top-ranked gene combinations as SL gene pairs (Alvarez et al., 2016; Hu et al., 2019), though neither statistical inference nor network-based methods are trained using known SL data.

In recent years, machine learning methods, such as the random forest (RF) algorithm, have gained popularity in the prediction of SL. Li and colleagues calculated enrichment scores from pathways included in the Gene Ontology (GO) and Kyoto Encyclopedia of Genes and Genomes (KEGG) databases for each gene as features in RF (Li et al., 2019), and DiscoverSL incorporated multi-omics data of cancer patients, including copy number variation (CNV), mutation, and expression, as input features for context-specific SL predictions using RF (Das et al., 2019). Moreover, SLant (synthetic lethal analysis *via* network topology) considers protein-protein interaction (PPI) and GO data as feature sources by manually calculating nodewise and pairwise network parameters and applying the RF algorithm to predict novel SL pairs (Benstead-Hume et al., 2019). Ensemble-based models, including MNMC (multi-network and multi-classifier) (Pandey et al., 2010), MetaSL (meta analysis of synthetic lethality) (Wu et al., 2014), and a model proposed by Lu’s research team (Lu et al., 2015), have been used to discover potential SL pairs, manually extracting features from either multiple biological networks (Pandey et al., 2010; Wu et al., 2014) or multi-omics data (Lu et al., 2015) and performing predictions based on a collection of classifiers. Mashup integrates multiple heterogeneous networks using graph representation learning methods, such as random walk with restart (RWR), to learn compact topological feature representations of genes and applies a support vector machine (SVM) to predict SL pairs (Cho et al., 2016). Collective matrix factorization (CMF) is another approach that integrates multiple heterogenous networks to learn latent representations for predicting SL interactions (Liany et al., 2020b). Similar to CMF, GRSMF (Huang et al., 2019) and SL^2^MF (Liu et al., 2020) are also matrix factorization (MF)-based methods, which adopt an encoder-decoder paradigm. These methods utilize different types of MF encoders, for example, graph regularized self-representative matrix factorization (Huang et al., 2019) or logistic matrix factorization (Liu et al., 2020), to decompose the SL matrix constructed from known SL gene pairs and then reconstruct the matrix using latent representations to predict novel SL pairs.

Nevertheless, MF-based methods are shallow embedding methods without sharing any parameters between nodes or leveraging node features, and this may limit their learning capacity. In contrast, graph neural networks (GNN) can effectively capture graph structures and learn informative embeddings by aggregating information from neighboring nodes. One widely used GNN model is the graph convolutional network (GCN) (Kipf and Welling, 2016). Cai and associates proposed DDGCN (dual-dropout GCN), the first GNN-based model to predict SL, which utilized dropout techniques to overcome overfitting and optimize prediction performance (Cai et al., 2020). Another GNN model, GCATSL (graph-contextualized attention network for predicting SL), integrated diverse biological sources (GO and PPI) as features input to improve SL prediction and included a graph attention network (GAT), a more advanced type of GNN, to learn node embeddings from multiple sources with different weights (Long et al., 2021). The other GNN model for SL prediction, KG4SL (knowledge graph neural network for synthetic lethality), integrates such factors as biological processes, diseases, and compounds that could be pertinent to SL interactions into a knowledge graph (KG) to facilitate useful interpretations (Wang et al., 2021). A recently proposed method, PiLSL (pairwise interaction learning-based GNN model), also considers knowledge graph as input features as well as omics features to predict novel SL gene pairs (Liu et al., 2022). It first constructs enclosing subgraphs for pairs of genes from the knowledge graph and then utilizes attentive embedding propagation to learn latent embeddings of the gene pair for the final prediction.

Though the performance of statistical inference, network models, machine learning, and GNN-based methods have been promising, their application still faces challenges. First and far most, all these approaches were designed to predict SL at the population level. The population-based SL prediction reflects that input omics data and features are derived from a set of cell lines or a collection of tumor samples from multiple patients, and these features are not designed for an individual sample. In the machine learning and GNN models, SL training and validation data were often collected from the SynLethDB database (Guo et al., 2016) in which SL prediction is not specific to an individual cell line. When applying these models for SL gene pair selection, its SL prediction lacks a context under specific cancer biology. In other words, current methods are limited to selecting common SL gene pairs among all cancer types. It cannot predict SL for a particular cancer cell. Second, none of these GNN methods have integrated multiple biological graph features when making predictions. They either only consider known SL network (DDGCN, GCATSL) without other graph features, or utilize knowledge graph that ignores individual information contained in different biological graphs (KG4SL, PiLSL).

Here, we propose a novel multi-view graph convolutional network model for the prediction of SL in individual cancer cells (MVGCN-iSL). Our model, MVGCN-iSL, comprises three parts. In the first, the GCN processes multiple biological networks independently as cell-specific and cell-independent input graphs to obtain graph-specific representations that provide diverse information for SL prediction. In the second part, a max pooling operation integrates several graph-specific representations into one, and in the third part, a multi-layer deep neural network (DNN) model utilizes these integrated representations as input to predict SL. Extensive experimental results demonstrate that MVGCN-iSL achieves state-of-the-art performance in the prediction of novel SL gene pairs as well as generalization to SL pairs of novel genes.

## 2 Materials and methods

### 2.1 Data collection

We collected cancer cell-specific SL data using the mapping system of Horlbeck and colleagues, who quantified genetic interactions of pairwise combinations of 472 genes in two cell lines (K562 and Jurkat) *via* a double-knockdown CRISPR (clustered regularly interspaced short palindromic repeats) interference (CRISPRi) technique (Horlbeck et al., 2018). Only those gene pairs with genetic interaction scores below -3 are considered SL gene pairs.

We collected multi-omics data, including gene expression, copy number, and mutation, from the Cancer Cell Line Encyclopedia (CCLE) database (Ghandi et al., 2019) and CRISPR essentiality data from the Cancer Dependency Map portal (DepMap) (Meyers et al., 2017), derived protein-protein physical interaction data and genetic interaction data from the Biological General Repository for Interaction Datasets (BioGRID) (Oughtred et al., 2019), and removed any genetic interactions that overlapped between BioGRID and Horlbeck’s mapping from BioGRID.

### 2.2 Input features

#### 2.2.1 Cell-specific networks

Informative cell-specific network features are generated from or dependent on the cell line in which we are predicting. In our model, based on known experimentally validated SL interactions in the specific cell line, we constructed a cell-specific SL graph in which each node represents a gene and each edge represents SL interaction. We consider this graph representative of the cell-specific network and that its topology can provide valuable information about unknown SL interactions within this specific cell line.

#### 2.2.2 Cell-independent networks

Apart from cell-specific networks, we also consider cell-independent network features that are derived from general population-based analysis and not specific to one cell line. Our model incorporates four types of cell-independent biological network features. We use the BioGRID database to generate two PPI networks, one for physical interactions and the other for genetic interactions, which together represent a union set of protein interactions from multiple different cell lines and reveal common relationships between genes. We then calculate Pearson correlation between each pair of genes based on CCLE expression profiles and build a co-expression network by connecting significant gene pairs (*p* < 0.01) in the network. Similarly, we build a co-essentiality network using DepMap CRISPR essentiality profiles. These cell-independent networks reflect some common patterns of interaction between genes that may offer valuable information for predicting synthetic lethality that is specific to one cell line.

#### 2.2.3 Gene node features

Apart from network features, initial representations for gene nodes, known as node features, are also crucial for training the model. These features include expression, copy number, and mutation derived from CCLE and essentiality derived from DepMap for each gene. They are cell-specific and provide additional information about the gene that may complement that from input biological networks.

### 2.3 Model speculation

**FIGURE 1:**
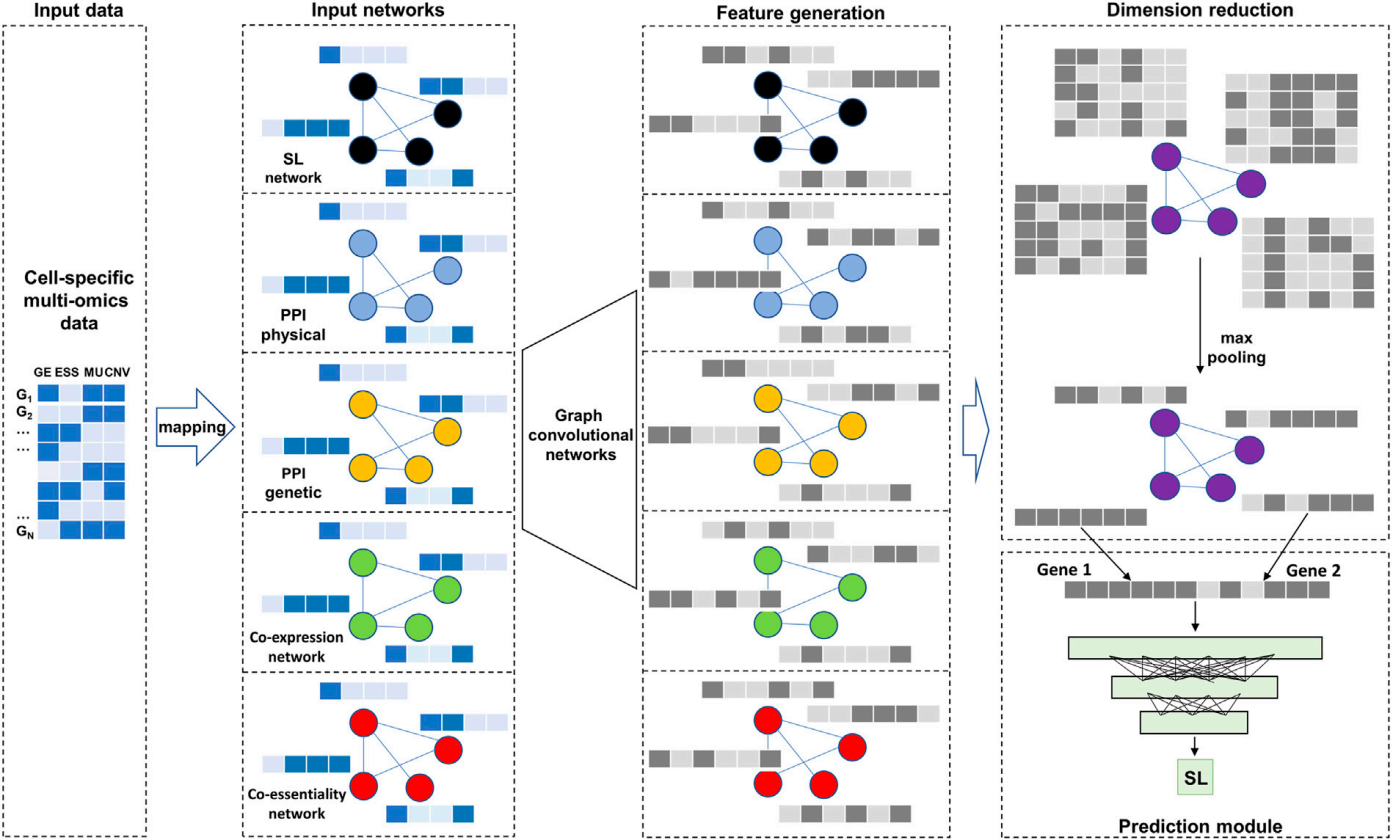
Overall model architecture. There are two types of input data: cell-specific multi-omics data as node features and five biological networks. After two layers of Graph convolutional networks and max pooling operation, the generated embeddings are used as input to a deep neural network for predicting SL.

Given multiple undirected graphs, $\left\{G^{\left(i\right)}=\left(V,\;E^{\left(i\right)}\right)\;:\;i\in \left\{1,2,3,4,5\right\}\right\}$ , there are $N=\left|V\right|$ nodes, i.e., genes. These five graphs are indexed from one to five, in the order of cell-specific SL graph, cell-independent physical PPI network, genetic interaction network, co-expression network, and co-essentiality network. The adjacency matrix $A^{\left(i\right)}$ is derived from known network information for each input graph and is symmetrically normalized after adding self-loops (Hall, 2010). The input node features form an N×R matrix X that contains four multi-omics features–gene expression, copy number, mutation, and essentiality (R = 4) for each gene node. In this work, we formulate the prediction of SL as a supervised classification task. Formally, given a set of known SL gene pairs, we incorporate multiple input graph features $G^{\left(i\right)}$ and node features X in an effort to predict whether novel gene pairs are SL pairs. Figure 1 depicts the overall architecture of our model, consisting of basic graph convolution operations applied independently over multiple graphs, the use of max pooling operations to integrate and the utilization of deep neural networks as the final module for the prediction of synthetic lethality.

### 2.4 Graph convolution

The core part of our MVGCN-iSL model is the graph convolution operation defined as (Kipf and Welling, 2016):

$$H^{\left(i\right)}=f\left(A^{\left(i\right)}XW^{\left(i\right)}\right)$$ (1)

where $W^{\left(i\right)}$ is the trainable weight matrix of the neural network for processing the $i^{th}$ input graph, $H^{\left(i\right)}$ is the updated feature matrix for the $i^{th}$ input graph, and f is an activation function, e.g., $ReLU\left(\cdot \right)=\max\left(0,\cdot \right)$ .

This graph convolution operation computes a node’s new features as the weighted average of its features and those of its neighbors (Li et al., 2018), naturally combining both graph structures and node features in the convolution. Through this aggregation scheme, two nodes with identical neighboring structures with identical node features on corresponding nodes will have identical representations (Xu et al., 2018). Therefore, the representation $H^{\left(i\right)}$ generated from the graph convolution operation is regarded as a good characterization of similarities based on both graph information and node features.

Furthermore, the graph convolution operation can be stacked into multiple layers to enable learning over a larger local neighborhood. However, using too many layers can mix node features over a long distance and make them indistinguishable (Li et al., 2018). Here, we adopted a two-layer model:

$$Z^{\left(i\right)}=f\left(A^{\left(i\right)}f\left(A^{\left(i\right)}XW_{0}^{\left(i\right)}\right)W_{1}^{\left(i\right)}\right)$$ (2)

where $W_{0}^{\left(i\right)}$ and $W_{1}^{\left(i\right)}$ are trainable weight matrices in the first and second graph convolutional layer for the $i^{th}$ input graph. The dimension of generated embeddings is determined by the number of neurons in the second graph convolutional layer, which is a predetermined number. Considering that all five GCN models have the same input node feature matrix X , we share trainable weight matrices across five GCN models to reduce the model complexity.

### 2.5 Integration of multiple graph features: Max pooling

In the former step, running the graph convolution operation in each input graph generated a set of five graph-specific representation matrices $\left\{Z^{\left(i\right)}:i\in \left\{1,2,3,4,5\right\}\right\}$ , where each $Z^{\left(i\right)}$ is of the dimension N×K . Here, K is set at 128 to generate a 128-length vector for each gene based on each input graph. To integrate these five hidden representation matrices, we utilize the max pooling operation, a popular technique in convolutional neural network models for processing images (Krizhevsky et al., 2012). Max pooling is a down-sampling strategy to reduce model parameters and control overfitting. The integration scheme is thus defined as:

$$Z_{jk}=\max\left\{Z_{jk}^{\left(i\right)}:i\in \left\{1,2,3,4,5\right\}\right\}$$ (3)

where $j\in \left[1,N\right]$ and $k\in \left[1,K\right]$ . Basically, we are taking the maximum value for each feature across five vectors and summarizing five N×K representation matrices into one final informative representation matrix. Practically, considering that some genes are only present in one or two graphs, gene representations generated from a graph that does not involve them will not be informative. Max pooling across multiple graphs highlights the more important role of the most influential graphs at an individual gene level.

### 2.6 Prediction module and optimization

After max pooling, the final representation matrix Z will generate integrated features for a gene pair as input for a prediction module. A three-layer deep neural network (DNN) model is introduced to serve as the prediction model. By stacking multiple layers, the DNN can learn an extremely intricate non-linear function mapping from the input to the output target, indeed, working best when the task is inherently non-linear, as indicated in this SL prediction task (Lecun et al., 2015). Because this is a binary classification task, the binary cross-entropy is used as the cost function:

$$L=-\frac{1}{M}\overset{M}{\underset{j=1}{\sum }}y_{j}\cdot \log\left(p\left(y_{j}\right)\right)+\left(1-y_{j}\right)\log\left(1-p\left(y_{j}\right)\right)$$ (4)

where M is the number of training samples, $y_{j}$ is the true label of the $j^{th}$ sample (0 or 1), $p\left(y_{j}\right)$ is the predicted probability of being SL by our model for the $j^{th}$ sample. The stochastic gradient descent (SGD) algorithm is applied to perform the optimization.

### 2.7 Comparison methods and evaluation metrics

We compared our MVGCN-iSL model with four state-of-the-art GNN-based models, DDGCN (Cai et al., 2020), GCATSL (Long et al., 2021), KG4SL (Wang et al., 2021) and PiLSL (Liu et al., 2022). DDGCN proposes a novel dual-dropout mechanism to solve the overfitting problem. It employs known SL gene pairs to construct an SL interaction network in which each gene is a node and SL interactions form edges. The dual dropout consists of dropouts of a coarse-grained node and a fine-grained edge. The node dropout involves the random dropping of some gene nodes in each training iteration, which forces the GNN model to learn more robust representations without overfitting. In the edge dropout, the random removal of some edges during the training enables further fine-tuning of the dropout at the edge level. However, the lack of external features limits the ability of DDGCN to generalize to novel genes without any known SL information.

GCATSL incorporates various biological data sources and utilizes a graph attention network (GAT). Compared to basic GNN models, GAT assigns different weight values to different neighbors to distinguish and preserve the difference among neighbors (Veličković et al., 2017). In GCATSL, three feature matrices are first constructed from biological processes (BP) and cellular components (CC) from Gene Ontology (GO) as well as the PPI network from the BioGRID database as input features. Then a dual-attention, i.e., node- and feature-level attention, mechanism is designed to learn node representations from multiple feature graphs. Specifically, node-level attention is used with GAT to learn preliminary representations for each input feature graph, and feature-level attention is implemented to integrate the representations learned from these three feature matrices to learn the importance of different feature inputs and generate the final representation for each gene node.

Both KG4SL and PiLSL incorporate knowledge graph as the input feature for predicting SL, considering that shared biological factors in knowledge graph might imply the dependency among SL gene pairs latently. KG4SL simply employs attention mechanism to learn different weights for different types of nodes and edges in each GNN layer, while PiLSL first constructs local enclosing subgraph of each gene pair and then utilize attention mechanism to learn latent embeddings for the gene pair in the subgraph. Besides, PiLSL integrates multi-omics data to further obtain powerful representations for more robust predictions.

We consider three evaluation metrics to compare SL prediction performance. The first two metrics, area under the receiver operating characteristic curve (ROC-AUC) and area under the precision-recall curve (AUPR), are threshold-free. The third metric, Precision@k, reflects the proportion of true positive samples in the top k% predictions to demonstrate our model’s ability to prioritize the top SL pairs. When comparing performance among population-based approaches, we consider the fourth metric, F-max, indicating the highest harmonic mean of precision and recall (F-measure) over all possible thresholds.

## 3 Results

### 3.1 Experimental setup

Our MVGCN-iSL model culminates with 128 and 64 neurons in the two graph convolutional layers and 64, 32, and 16 neurons in the three-layer deep neural network. The model is optimized by the Adam optimizer with the learning rate of 0.0001 (Kingma and Ba, 2014). Early stopping technique is utilized to reduce over-fitting. MVGCN-iSL is implemented using the PyTorch Geometric library in Python and takes advantage of the powerful computing capacity of multiple graphic processing units (GPUs) (Fey and Lenssen, 2019). We carried out all experiments on the Pitzer cluster provided by the Ohio Supercomputer Center (OSC) with central processing units (CPU) of 48 cores and 192 GB RAM. The GPUs used were two NVIDIA^®^ Tesla V100 GPUs with 32 GB RAM. The implementation of MVGCN-iSL is available at https://github.com/kunjiefan/MVGCNiSL.

We conduct experiments on two cancer cell-lines (K562 and Jurkat) individually. For the K562 cell line, 1,523 of 100,128 samples (1.5%) are SL gene pairs, whereas only 373 of 74,691 samples (0.5%) in the Jurkat cell line are SL gene pairs. We consider Precision@5 metric for K562 cell line while use Precision@10 for Jurkat cell line given limited positive samples. We split the dataset into an 80% training set and a 20% test set, performed five-fold cross-validation on the training set to determine hyper-parameters, and evaluated model performance based on the test set. During the training, at each epoch we randomly sampled some of the negative samples to ensure a balanced training set.

We consider two evaluation settings: leave-gene-combination-out and leave-gene-out. Under leave-gene-combination-out setting, training and test data are completely randomly sampled, where both genes of a pair in the test set might be present in the training set. As for leave-gene-out setting, we first randomly split genes into training and test, and use gene pairs within as training and test set, respectively. The leave-gene-combination-out setting evaluates a model’s ability to complete missing SL data within a set of selected genes of interest when only part of the interactions is known, while leave-gene-out measures the ability of the model to generalize to SL gene pairs of novel genes with no available data.

### 3.2 Population-based SL prediction approaches cannot predict cell-specific SL gene pairs

We compared prediction performance between two population-based methods, DAISY and a second population-based model denoted as “Population” and a random model, examining precision, recall, and f-max to determine the suitability of these methods for cell-specific SL prediction under leave-gene-combination-out evaluation setting in K562 cell line (Table 1). We extracted predictions of DAISY, a statistical-inference method that uses multi-omics data from a collection of cancer cell lines without considering cell-specific features (Jerby-Arnon et al., 2014), from SynLethDB (Guo et al., 2016) and calculated precision, recall, and f-max based on the overlapping of data with that of Horlbeck’s mapping (Horlbeck et al., 2018). For the “Population” model, which uses the co-expression network as the input graph and gene expression profiles from CCLE as node features, we utilized principal component analysis (PCA) to reduce the dimensionality of CCLE gene expression profiles to four to be consistent with our cell-specific model. Our random model utilized a randomly generated network and randomly generated node features as input features.

**TABLE 1:** Performance comparison between population-based methods and a random model. The evaluation is under leave-gene-combination-out setting in K562 cell line. The random model uses randomly generated network and randomly generated gene features as inputs.

	Precision	Recall	F-max
DAISY	0.541	0.003	0.005
Population	0.554	0.938	0.693
Random	0.633	0.832	0.692

As shown in Table 1, neither of our two population-based models performed better than the random model. DAISY performed extremely poorly, indicating that an unsupervised population-based model is not suitable for cell-specific SL prediction, and though our “Population” model yielded recall of 0.93, its precision of only 0.55 resulted in an F-max of 0.69. All these results highlight drawbacks of using population-based SL prediction models for cell-specific SL prediction and the importance of developing cell-specific prediction models.

### 3.3 Integration of multiple molecular networks improves prediction performance

**FIGURE 2:**
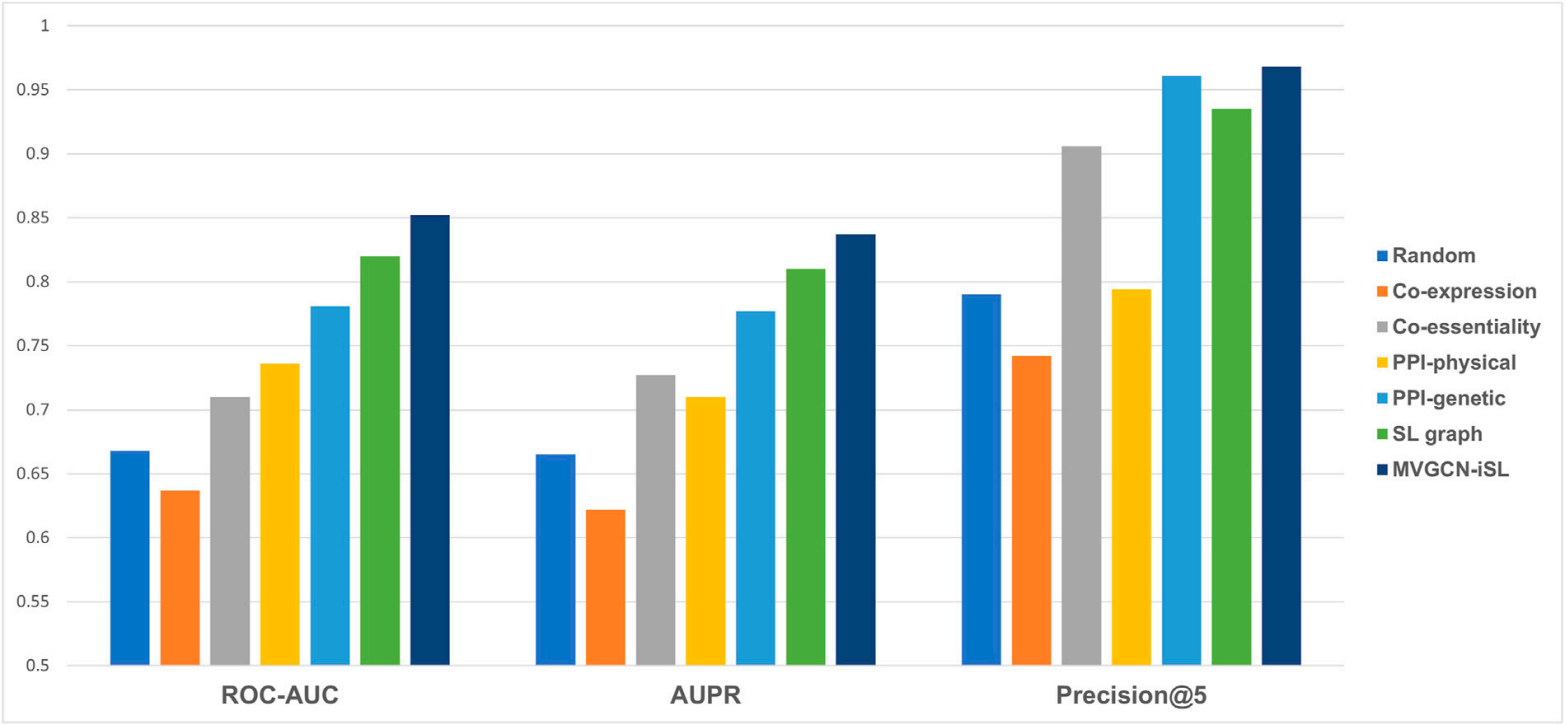
Model performance comparison across different types of input molecular networks under leave-gene-combination-out setting in K562 cell line. Performance is compared among five different input molecular networks, a random model and our final model, MVGCN-iSL, in terms of three evaluation metrics: ROC-AUC, AUPR and Precision@5. “Random” is a model using randomly generated network as the input.

Our MVGCN-iSL model employed five molecular network graphs and gene node features to predict SL gene pairs (Figure 2). Though cell-independent network features are not informative for predicting cell-specific SL pairs, the information they imply about common synthetic lethality across cells might serve to complement cell-specific network features and improve prediction when integrated with cell-specific features. In addition, we designed a random model that utilized a randomly generated network as the input graph in combination with cell-specific node features, which served as a base model. All experiments in this section were conducted under leave-gene-combination-out setting in K562 cell line.

As illustrated in Figure 2, none of the three metrics reflected better performance of the co-expression network than the random model, while co-essentiality, physical PPI network, genetic PPI network and SL graph all showed all-around improvement over the base model. Among these five molecular network features, SL graph and genetic PPI network demonstrate the best performance. The Co-essentiality network feature also shows promising results, especially in terms of Precision@5 with a value of 0.906. Taken together, our model, MVGCN-iSL, that combines all five graphs, yielded a ROC-AUC of 0.852, AUPR of 0.837, and Precision@5 of 0.968, indicating that the integration of multiple molecular networks improves prediction performance. When comparing the failed cases with correctly predicted cases of our MVGCN-iSL model, we found that genes in those failed cases have a higher chance of missing in one or more input graphs. Especially, when one or both genes in the pair are missing in the genetic interaction network or co-essentiality network that have been proved to be important for SL prediction, this pair is more likely to be incorrectly predicted. This analysis verified the importance of incorporating multiple sources of biological networks, since remaining networks can still contribute to the prediction when genes are missing in some input networks.

### 3.4 MVGCN-iSL outperforms existing GNN methods

We compared prediction performance of our MVGCN-iSL model with that of four existing GNN methods, DDGCN (Cai et al., 2020), GCATSL (Long et al., 2021), KG4SL (Wang et al., 2021) and PiLSL (Liu et al., 2022), which have been demonstrated to achieve state-of-the-art performance in predicting population-based SL gene pairs using the SynLethDB database. We made comparisons under two evaluation settings (leave-gene-combination-out and leave-gene-out) in two cell-specific datasets (K562 and Jurkat). Although these approaches were initially designed for predicting population-based SL interactions, they were adapted to predict cell-specific SL gene pairs.

Under leave-gene-combination-out setting, as shown in Table 2, we can see that DDGCN performs the worst in both two cell lines, indicating that external features are required for the prediction. KG4SL is not achieving promising results as well, which might demonstrate that the use of knowledge graph is not suitable for cell-specific SL prediction. All evaluation metrics depict the superior prediction performance of MVGCN-iSL to that of GCATSL. The primary difference between MVGCN-iSL and GCATSL is how the model uses multiple graph features. MVGCN-iSL utilizes graph structure information directly and integrates their data through a max pooling operation. In contrast, GCATSL transforms graph information into feature maps by calculating pairwise similarity and integrates multiple feature maps together as the input node features in the GNN model. Thus, it seems that the direct use of graph structures yields better results than the derivation of node features from the graph. Compared to PiLSL, MVGCN-iSL achieves better performance across all metrics in K562 cell line and gets comparable results in terms of AUPR and Precision@10 in Jurkat cell line. Though PiLSL demonstrates promising results, it typically takes 20x more computing time to train the model than MVGCN-iSL, which greatly limits its applicability in practice.

**TABLE 2:** Comparison with four GNN methods in two cell-specific datasets under leave-gene-combination-out evaluation setting.

Model	K562			Jurkat		
	ROC-AUC	AUPR	Precision@5	ROC-AUC	AUPR	Precision@10
DDGCN	0.631	0.669	0.954	0.536	0.597	0.781
GCATSL	0.812	0.803	0.912	0.752	0.771	0.867
KG4SL	0.734	0.723	0.923	0.695	0.684	0.723
PiLSL	0.831	0.763	0.839	0.807	0.820	0.972
MVGCN-iSL	0.852	0.837	0.968	0.825	0.819	0.967

As for leave-gene-out setting (Table 3), which evaluates the ability of the model to generalize to SL gene pairs of novel genes with no available data, the comparison results display similar patterns as the leave-gene-combination-out setting. Notably, DDGCN is not able to predict SL for genes without known SL information, since it only relies on SL network constructed from existing data. Both GCATSL and KG4SL show poor results in terms of all metrics, indicating inability to generalize to novel genes. Compared to PiLSL, MVGCN-iSL obtains higher performance across all metrics in K562 cell line and higher AUPR in Jurkat cell line, with slightly lower ROC-AUC and Precision@10 in Jurkat cell line. Taken together, MVGCN-iSL has achieved state-of-the-art performance under both leave-gene-combination-out and leave-gene-out settings.

**TABLE 3:** Comparison with four GNN methods in two cell-specific datasets under leave-gene-out evaluation setting.

Model	K562			Jurkat		
	ROC-AUC	AUPR	Precision@5	ROC-AUC	AUPR	Precision@10
DDGCN	-	-	-	-	-	-
GCATSL	0.523	0.516	0.528	0.508	0.552	0.521
KG4SL	0.508	0.508	0.515	0.501	0.505	0.318
PiLSL	0.627	0.616	0.611	0.629	0.608	0.667
MVGCN-iSL	0.642	0.623	0.632	0.596	0.643	0.598

### 3.5 MVGCN-iSL is robust under small sample sizes

When compared with population-based prediction, the lack of SL training data in a specific cell line presents a primary challenge in the prediction of cell-specific SL gene pairs. Practically speaking, an ideal model could achieve promising results even with a relatively small training sample size. With this in mind, we conducted a series of experiments to evaluate the performance of our model using different numbers of training samples (10%, 30%, 50%, 70% of total samples) under leave-gene-combination-out setting in K562 cell line as shown in Table 4. The process of splitting data into training and test set is completely random, no matter the proportion.

**TABLE 4:** Performance comparison with different training sample sizes. The evaluation is under leave-gene-combination-out setting in K562 cell line dataset. The proportion column indicates the proportion of total samples used as training samples.

Proportion	ROC-AUC	AUPR	Precision@5
0.1	0.745	0.747	0.903
0.3	0.785	0.774	0.914
0.5	0.820	0.807	0.935
0.7	0.844	0.832	0.957

We expected the model’s performance to improve in all metrics as the number of training samples increased. The results for Precision@5 show that prediction performance of all four models exceeded 0.9, which is a very promising result. More specifically, when we only consider 10% of the total samples in the training set (∼150 SL gene pairs), out of the top 5% predictions with the highest confidence, 90.3% of predicted gene pairs are true SL gene pairs. This data shows significant applicability in the prioritization of SL gene pairs for biologists.

## 4 Discussion and conclusion

MVGCN-iSL is a multi-view GNN model that incorporates five distinct biological graphs and cell-specific multi-omics data to predict cell-specific SL gene pairs. The powerful representation capability of the GNN and integration of multiple informative features allow our model to consistently outperform existing state-of-the-art models. Notably, high Precision@5 score of our model even with a limited number of training samples demonstrates its applicability for the prioritization of experiments for cell-specific SL validation.

Among the five input graph features, the co-expression and co-essentiality networks make totally different contributions though they are generated in a similar way (Figure 2), and essentiality features seem much more informative than expression features. To investigate any associations between synthetic lethality and expression or essentiality, we calculated Spearman’s rank correlation between the median of SL values and essentiality scores or expression values separately and observed negative correlation between SL and essentiality (−0.19, *P* < 1e-4) and no correlation between SL and expression (0.03, *p* = 0.537). This explains why essentiality is more helpful than expression for predicting SL. This negative correlation implies the reduced likelihood that a gene with greater essentiality will be synthetic lethal with other genes, which is consistent with the definition of synthetic lethality.

One limitation of our current model is the lack of more cell-specific input graph features. Currently, we only include a cell-specific SL graph that we have shown to be the most informative. In the future, we will try to incorporate more cell-specific graphs, for example, building a cell-specific co-expression network based on perturbation data in the Library of Integrated Network-based Cellular Signatures (LINCS) database (Subramanian et al., 2017). Another future direction is to build a model that can directly predict SL on a novel cell line without first training on that cell line. Assuming the existence of some common underlying mechanisms of SL among different cell lines, it is possible that we can train a model based on one cell line and then use the trained model to predict SL directly on another cell line *via* transfer learning. This type of model can aid biologists in accelerating the process of selecting genes for experimental validation in a novel unexplored cell line.

## Data Availability

The datasets presented in this study can be found in online repositories. The names of the repository/repositories and accession number(s) can be found below: https://github.com/kunjiefan/MVGCNiSL.
